# Sanguineous cardiopulmonary bypass prime accelerates the inflammatory response during pediatric cardiac surgery

**DOI:** 10.1177/02676591241291944

**Published:** 2024-10-09

**Authors:** Joel Bierer, Roger Stanzel, Mark Henderson, John Sapp, Pantelis Andreou, Jean S. Marshall, David Horne

**Affiliations:** 1 Division of Cardiac Surgery, 3688 Dalhousie University, Halifax, NS, Canada; 2 Department of Clinical Perfusion, 432234 Nova Scotia Health Authority, Halifax, NS, Canada; 3 Division of Cardiology, 3688 Dalhousie University, Halifax, NS, Canada; 4 Department of Community Health & Epidemiology, 3688 Dalhousie University, Halifax, NS, Canada; 5 Department of Microbiology & Immunology, 3688 Dalhousie University, Halifax, NS, Canada

**Keywords:** perfusion, cardiopulmonary bypass, pediatric cardiac surgery, congenital heart disease, complement, inflammation

## Abstract

**Background:**

The inflammatory response to cardiopulmonary bypass (CPB) in pediatric patients remains an unresolved challenge. Sanguineous CPB prime, composed of allogenic blood products, is one potentially important stimulus. This study aims to identify specific inflammatory mediators active in sanguineous CPB prime and their impact on the inflammatory response at CPB initiation.

**Methods:**

In a post-hoc analysis of a prospective observational cohort study (NCT05154864), where pediatric patients undergoing cardiac surgery with CPB were enrolled after informed consent, patients were grouped by CPB prime type (*sanguineous* vs *crystalloid*). Arterial samples were collected post-sternotomy as a baseline and again at CPB initiation from all patients. In the *sanguineous* group, CPB prime samples were also collected after buffered ultrafiltration but before CPB initiation. *Luminex®* measured concentrations of 24 inflammatory mediators for comparison between groups. Statistical analyses were by Mann-Whitney test and Wilcoxon signed-rank test. Data are presented as median [IQR].

**Results:**

Forty consecutive pediatric patients participated. The *sanguineous* group (*n* = 26) was younger (4.0 [0.2 – 6.0] vs 48.5 [39.0 – 69.5] months; *p* = 2.6 × 10^−7^) and smaller (4.9 [34 – 6.6] vs 17.2 [14.9 – 19.6] kg; *p* = 2.6 × 10^−7^) than the *crystalloid* group (*n* = 14). Despite this, baseline concentrations of 20 complement and cytokine concentrations were comparable between groups (*p* > 0.05) while four showed differences between groups (*p* < 0.05). The sanguineous prime contained supraphysiologic concentrations of complement mediators: C2, C3, C3a, C3b, and C5a. Correspondingly, upon CPB initiation, patients receiving sanguineous prime exhibited a significantly larger burden of C2, C3, C3b, C5, and C5a (*p* < 0.001) relative to the crystalloid group. Cytokine and chemokine mediators were present at trace levels in the sanguineous prime.

**Conclusions:**

Sanguineous prime contains activated complement that accelerates the inflammatory response at CPB initiation in neonates and infants. Immunomodulatory interventions targeting complement during CPB prime preparation could offer substantial benefits for these vulnerable patients.

## Background

Cardiopulmonary bypass (CPB) stimulates an innate inflammatory response during pediatric cardiac surgery which impacts the patient’s post-operative recovery.^1–4^ The patient’s age, weight, pathology, CPB time, ischemia-reperfusion, blood transfusion, and hypothermia contribute to the noxious response and organ dysfunction.^2,3,5^ Accordingly, neonates and small infants experience the highest rates of morbidity and mortality after congenital cardiac surgery due to the nature of their pathology and peri-operative factors.^6^ Sanguineous CPB prime is commonly used for these small patients to prevent excessive hemodilution and biochemistry disturbance upon CPB initiation, maintain adequate oxygen delivery during the surgery, and manage coagulopathy.^7–9^ Unfortunately, achieving these objectives comes at the cost of substantial exposure to allogenic blood products which carry pro-inflammatory stimuli and red blood cell storage lesions including oxidized hemoglobin components, toxic cell-free hemoglobin, lactic acid, and hyperkalemia.^10^ The fragile reconstituted blood is exposed to the non-endothelialized CPB circuit for some time prior to perfusing the patient, theoretically aggravating its toxic potential by the contact and complement system activation.

Preparing a physiologic sanguineous prime with a normal acid-base, electrolytes, and reduced inflammatory burden has been an area of interest for decades.^11–13^ Outside of standard blood collection, preparation, and storage procedures, buffered ultrafiltration of the prime (BUF) has been adopted to accomplish these important goals. Clinical studies in neonates and small infants suggest that this technique, commonly termed “washing”, reduces inflammatory mediator concentrations in the prime which translates to dampened inflammatory reaction during CPB initiation and expedited recovery post-operatively.^11–14^ Unfortunately, these studies only assessed a limited number of inflammatory mediators in the context of a complex immunologic reaction to CPB and cardiac surgery.

The complement system has been established as the primary mechanism of CPB-associated inflammation. Recent research has suggested the degree of complement activation and production of anaphylatoxins C3a and C5a are most relevant to clinical inflammation, organ dysfunction and prolonged recovery after children’s heart surgery.^2,3,15^ The sanguineous prime contribution to CPB-associated inflammation remains largely unexplored, presenting a potential therapeutic opportunity to dampen inflammation and improve recovery after pediatric cardiac surgery. Accordingly, this investigation has two objectives. First, to identify the relevant inflammatory mediators present in reconstituted sanguineous CPB prime and, second, to examine their effect on the patient’s immune response upon the initiation of CPB.

## Methods

### Study design

This is a post-hoc cohort analysis using data from a single-center single-arm prospective clinical trial (NCT05154864 on ClinicalTrials.gov) investigating the relationship between circulating inflammatory mediators and post-operative clinical outcomes, in pediatric patients undergoing cardiac surgery with CPB and continuous ultrafiltration. In this report, the exposure of interest, is CPB prime composition – sanguineous versus crystalloid – for pediatric cardiac surgery. The outcomes of interest are inflammatory mediators (compliment, cytokines and chemokines) upon CPB initiation. Comparative analysis was conducted between *sanguineous* and *crystalloid prime* groups. Further, a within-individual pairwise analysis examined the inflammatory mediator dynamic changes over time. The study is reported following the Strengthening the Reporting of Observational Studies in Epidemiology (STROBE) guidelines (Supplement Figure 1).^16^

### Data sources

Written informed consent was obtained from substitute decision-makers for all participants under a protocol approved by the IWK Health Centre Research Ethics Board (#1024869) on November 21, 2019. Patients were prospectively enrolled and completed the study protocol between August 2020 and June 2021 at the IWK Health Centre in Halifax, Nova Scotia, Canada. Patient baseline demographics and intra-operative data were collected. Arterial blood (1 mL) was drawn post-sternotomy but prior to CPB initiation (Pre-CPB baseline), and another 1 mL of arterial blood was drawn once full-flow CPB was reached or at 5 min of CPB exposure, whichever came first (CPB initiation). In the *sanguineous prime* group, 1 mL of circuit CPB-prime was collected after BUF but before CPB initiation. All biologic samples were analyzed for inflammatory mediator concentrations, described below.

### Study participants

Patients weighing less than 30 kg undergoing congenital cardiac surgery with CPB were prospectively assessed for enrollment. Exclusion criteria included: absence of written consent, known severe hematologic abnormality, genetic syndrome with severe multi-organ involvement, immunodeficiency syndrome and severe liver disease. A total of 49 consecutive patients were screened during the study period and 40 patients (82%) gave informed consent to participate. Nine patients (18%) were not enrolled due to a genetic syndrome with severe multi-organ abnormalities (*n* = 3, 6%), weight over 30 kg (*n* = 3, 6%), logistical process restraints (*n* = 2, 4%) and refusal to participate (*n* = 1, 2%). All 40 enrolled patients completed data collection and are included in this post-hoc analysis with no exclusions.

### CPB prime preparation

CPB prime composition was according to our institution’s standard of care. Sanguineous prime – consisting of 200 mL packed red blood cells (RBC), 200 mL fresh frozen plasma (FFP), 300 mL of Plasma-Lyte® A and 300 mL of 0.45% saline – was used for patients <10 kg. The mixture underwent BUF to normalize metabolic parameters and hemoconcentrate the volume to approximately 500 mL.^17^ The crystalloid prime was used for those >10 kg and included 1000 mL Plasma-Lyte® A and 500 mL 5% albumin, which was reduced to roughly 700 mL. Retrograde autologous priming was considered for patients in the crystalloid group. Heparin, sodium bicarbonate, calcium chloride, magnesium sulfate, furosemide and mannitol were applied at standard weight-based doses. A *Liva Nova S5*^*TM*^ CPB System with phosphorylcholine coating (48-40-00, London, UK), *Terumo FX05* (weight <10 kg) or *FX15* (weight >10 kg) oxygenators (1CX*FX05RE/1CX*FX15 E, Tokyo, Japan) and *Terumo Capiox®* Hemoconcentrator HCO5 (1CX*HC05S, Tokyo, Japan) were used. The *Terumo FX05* (estimated surface area of 0.5 m^2^) has roughly one third the contact interface relative to the *Terumo FX15* (estimated surface area of 1.5 m^2^).

### Immunoanalysis

Arterial blood samples were collected in EDTA tubes, centrifuged for 10 min (0.5 × gravity), and the resulting plasma was extracted. The plasma underwent a second centrifugation for 20 min (2.5 × gravity) to yield a platelet-free plasma which was aliquoted, flash-frozen in liquid nitrogen and stored at −80°C. *Luminex* immunoanalysis of a panel of relevant mediators was completed with a *Bio-Rad Bio-Plex® 200* System (Hercules, United States). Twenty-four human inflammatory factors from the complement, cytokine and chemokine families – C1q, C2, C3, C3a, C3b, C4, C4b, C5, C5a, CFB, CFH, CFI, TNF, IL-1α, IL-1β, IL-1Ra, IL-6, IL-10, CCL2, CCL3, CCL4, CXCL1, CXCL2 and CXCL-8/IL-8) – were analyzed using multiple analysis kits including: *ThermoFisher* C3a Simplex Kit (EPX010-12,282-901, Waltham, United States), *Millipore Sigma* Human Complement Magnetic Beat Panel 1 (HCMP1MAG-19K-05, Burlington, United States), *Millipore Sigma* Human Complement Magnetic Beat Panel 2 (HCMP2MAG-19K-06, Burlington, United States), and *BioTechne R&D Systems* Human XL Cytokine Luminex Performance Panel (FCSTM18-21, Minneapolis, United States). Bio-Rad Bio-Plex® ManagerTM Software 6.2 (Hercules, United States) was used to complete the data acquisition and used Logistic - 5PL regression for all analytes.

### Statistical analysis

Categorical variables are reported as numbers (%), and continuous variables are presented as median (interquartile range). Differences in patient baseline demographics and baseline inflammatory mediator concentrations between *sanguineous* and *crystalloid* groups were evaluated by Mann-Whitney test and z-test for proportions. The difference in inflammatory mediator concentration between Pre-CPB baseline and CPB initiation was assessed in a paired fashion with Wilcoxon signed-rank test, and the median difference (MD) [95% confidence interval] was calculated by the exact permutation probability technique. The magnitude of mediator concentration difference between sanguineous CPB prime and patient Pre-CPB baseline was expressed as a median fold difference ([prime] – [baseline] / [baseline]) with the [95% confidence interval] estimated by 1000 non-parametric bootstrap samples. The within-individual magnitude of mediator concentration change upon CPB initiation relative to Pre-CPB baseline was expressed as a median fold change ([CPB initiation] – [baseline] / [baseline]) with the [95% confidence interval] estimated by 1000 non-parametric bootstrap samples. The inflammatory mediator concentrations were assumed to be 0 in the crystalloid prime. There were no subgroup analyses or loss to follow-up or sensitivity analyses. All statistical analyses were performed using R (version 4.2.3, *R Foundation for Statistical Computing*, Vienna, Austria). Statistical significance was alpha = 0.05.

## Results

### Participant demographics

Forty pediatric patients were analyzed as a total cohort; 26 patients received sanguineous CPB prime, and 14 patients received crystalloid CPB prime. Baseline characteristics of the group are summarized in Table 1. The *sanguineous prime* patients were significantly younger, had a lower weight, had a higher proportion of STAT three and four pathologies but no difference relative to male sex, compared to the *crystalloid prime* group. The Pre-CPB baseline concentrations of C1q, C2, C3, C3a, C3b, C4, C5, C5a, CFB, CFH, TNF, IL-1α, IL-1β, IL-10, CCL2, CCL3, CCL4, CXCL1, CXCL2 and CXCL8 were comparable between groups while the patients in the *sanguineous prime* group had statistically lower Pre-CPB baseline concentrations of C4b (11 [10 – 13] ug/ml vs 14 [12 – 15] ug/ml), CFI (17 [13 – 21] ug/ml vs 35 [27 – 46]) and higher concentrations of IL-6 (13 [9 – 29] pg/ml vs 6 [1 – 12] pg/ml) and IL-1Ra (468 [327 – 812] pg/ml vs 338 [281 – 365] pg/ml) relative to patients in the *crystalloid* group.

**Table 1.** Patient demographics.

	Sanguineous prime (*n* = 26)	Crystalloid prime (*n* = 14)	*p*-value	Cohort (*n* = 40)
Age (months)	4.0 (0.2 – 6.0)	48.5 (39.0 – 69.5)	2.6 × 10^−7^	7.5 (1.7 – 39.0)
Neonate	10 (39%)	0 (0%)	3.6 × 10^−7^	10
Infant	12 (46%)	0 (0%)		12
Child	4 (15%)	14 (100%)		18
Male sex	17 (63%)	6 (43%)	0.09	23 (58%)
Weight (kg)	4.9 (3.4 – 6.6)	17.2 (14.9 – 19.6)	2.6 × 10^−7^	6.7 (4.6 – 14.9)
Body surface area (m^2^)	0.29 (0.22 – 0.35)	0.74 (0.4 – 0.85)	3.5 × 10^−7^	0.35 (0.27 – 0.64)
STAT score				
STAT 1	6 (23%)	7 (50%)	1.5 × 10^−3^	13 (33%)
STAT 2	5 (19%)	7 (50%)		12 (30%)
STAT 3	2 (8%)	0 (0%)		2 (5%)
STAT 4	13 (50%)	0 (0%)		13(32%)
Inflammatory mediators at baseline				
C1q (ug/ml)	47 (34 – 70)	51 (35 – 76)	0.55	47 (34 – 73)
C2 (ug/ml)	0.5 (0.4 – 0.6)	0.3 (0.3 – 0.5)	0.11	0.4 (0.3 – 0.6)
C3 (ug/ml)	16 (10 – 26)	14 (9 – 18)	0.22	15 (10 – 23)
C3a (ng/ml)	19 (9 – 27)	17 (9 – 23)	0.70	17 (9 – 26)
C3b (ug/ml)	17 (1 – 42)	2 (2 – 19)	0.48	7 (2 – 28)
C4 (ug/ml)	145 (107 – 199)	193 (110 – 224)	0.44	155 (106 – 218)
C4b (ug/ml)	11 (10 – 13)	14 (12 – 15)	5.2 × 10^−3^	12 (10 – 14)
C5 (ug/ml)	13 (9 – 15)	13 (10 – 15)	0.71	13 (10 – 15)
C5a (pg/ml)	19 (11 – 63)	22 (7 – 80)	1.0	20 (8 – 73)
CFB (ug/ml)	142 (98 – 166)	160 (126 – 192)	0.35	151 (99 – 186)
CFH (ug/ml)	171 (119 – 257)	226 (150 – 260)	0.46	176 (129 – 262)
CFI (ug/ml)	17 (13 – 21)	35 (27 – 46)	1.3 × 10^−4^	20 (15 – 28)
TNF (pg/ml)	32 (22 – 38)	45 (20 – 35)	0.26	29 (22 – 37)
IL-1α (pg/ml)	55 (44 – 67)	59 (39 – 62)	0.74	58 (43 – 65)
IL-1β (pg/ml)	8 (5 – 10)	7 (5 – 9)	0.88	8 (5 – 10)
IL-6 (pg/ml)	13 (9 – 29)	6 (1 – 12)	0.02	10 (5 – 23)
IL-10 (pg/ml)	88 (35 – 128)	52 (40 – 94)	0.34	77 (38 – 113)
IL-1Ra (pg/ml)	468 (327 – 812)	338 (281 – 365)	0.01	367 (299 – 557)
CCL2 (pg/ml)	218 (161 – 265)	196 (182 – 237)	0.74	202 (167 – 262)
CCL3 (pg/ml)	33 (28 – 37)	29 (25 – 38)	0.36	31 (27 – 37)
CCL4 (pg/ml)	493 (461 – 583)	600 (493 – 733)	0.12	519 (467 – 644)
CXCL1 (pg/ml)	172 (148 – 210)	162 (134 – 264)	1.0	171 (135 – 220)
CXCL2 (ng/ml)	1.9 (1.2 – 2.6)	2.3 (1.2 – 3.0)	0.49	2.0 (1.1 – 2.7)
CXCL8 (pg/ml)	12 (10 – 23)	9 (8 – 11)	0.06	11 (9 – 18)

C: complement; CF: complement factor; CCL: C-C motif chemokine ligand; CXCL: C-X-C motif chemokine ligand; CPB: cardiopulmonary bypass; IL: interleukin; STAT: Society of Thoracic Surgeons-European Association for Cardio-Thoracic Surgery; TNF: tumor necrosis factor.

### Sanguineous prime composition

**Figure 1.**
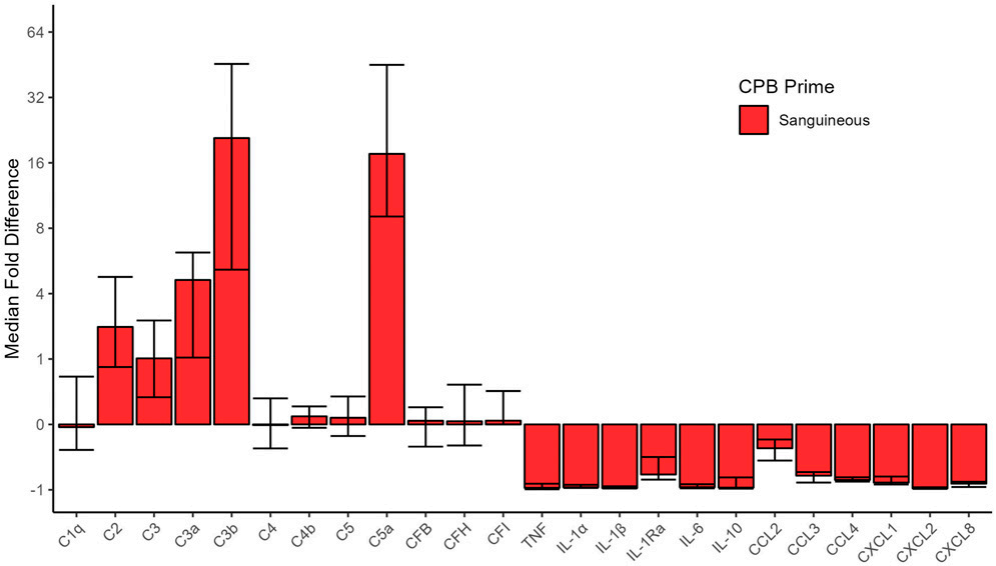
Fold difference [95% CI] of inflammatory mediators in the sanguineous CPB prime relative to patient pre-CPB baseline. Complement factors, but not cytokines or chemokines, in the sanguineous prime were generally found to be in significant excess relative to patient baseline. C: complement; CF: complement factor; CCL: C-C motif chemokine ligand; CXCL: C-X-C motif chemokine ligand; IL: interleukin; TNF: tumor necrosis factor.

The biochemical characteristics between sanguineous and crystalloid prime are inherently different (Table 2). Relative to the patient’s baseline, the sanguineous prime contained supraphysiologic complement levels of C2 (median fold difference of 2.8 [95% CI: 1.8 – 4.8] times), C3 (median fold difference of 1.3 [95% CI: 0.4 – 3.0] times), C3a (median fold difference of 4.6 [95% CI: 2.0 – 6.2] times), C3b (median fold difference of 20.8 [95% CI: 5.2 – 45.6] times) and C5a (median fold difference of 17.6 [95% CI: 9.1 – 45.2] times). C1q, C4, C4b, C5, CFB, CFH and CFI were not different between the patient baseline and sanguineous prime. The sanguineous prime had insignificant levels of all cytokines and chemokines, far lower than the patient’s baseline (Supplement Table S1). Figure 1 illustrates the fold difference of inflammatory mediators in the sanguineous prime relative to patient Pre-CPB baseline.

**Table 2.** Cardiopulmonary bypass prime characteristics.

	Sanguineous prime (*n* = 26)	Crystalloid prime (*n* = 14)	*p*-value
Prime volume (ml)	500 (450 – 638)	742 (575 – 852)	1.6 × 10^−3^
Prime volume (ml/kg)	102 (80 – 150)	39 (34 – 46)	8.4 × 10^−9^
BUF effluent volume (ml)	450 (312 – 600)	NR	NA
Post-BUF hemoglobin (g/L)	107 (92 – 126)	0	NA
Post-BUF acid-base			NA
pH	7.38 (7.33 – 7.41)	NR	NA
CO_2_ (mmHg)	48 (46 – 49)	NR	NA
HCO_3_ (mM)	26 (23 – 30)	NR	NA
Lactate (mM)	2.2 (1.6 – 3.0)	NR	NA
Inflammatory mediators			
C1q (ug/ml)	49 (24 – 76)	0	NA
C2 (ug/ml)	1.6 (1.1 – 2.3)	0	NA
C3 (ug/ml)	43 (28 – 67)	0	NA
C3a (ng/ml)	79 (52 – 123)	0	NA
C3b (ug/ml)	133 (57 – 291)	0	NA
C4 (ug/ml)	156 (96 – 198)	0	NA
C4b (ug/ml)	13 (11 – 16)	0	NA
C5 (ug/ml)	11 (9 – 18)	0	NA
C5a (pg/ml)	584 (267 – 1605)	0	NA
CFB (ug/ml)	130 (75 – 161)	0	NA
CFH (ug/ml)	180 (96 – 297)	0	NA
CFI (ug/ml)	22 (16 – 34)	0	NA
TNF (pg/ml)	0.9 (0.2 – 3.3)	0	NA
IL-1α (pg/ml)	2.1 (1.2 – 6.0)	0	NA
IL-1β (pg/ml)	0.2 (0.1 – 0.6)	0	NA
IL-6 (pg/ml)	0.6 (0.3 – 1.5)	0	NA
IL-10 (pg/ml)	3.6 (1.3 – 9.4)	0	NA
IL-1Ra (pg/ml)	155 (105 – 279)	0	NA
CCL2 (pg/ml)	155 (90 – 187)	0	NA
CCL3 (pg/ml)	5.6 (3.1 – 9.7)	0	NA
CCL4 (pg/ml)	90 (67 – 107)	0	NA
CXCL1 (pg/ml)	21 (13 – 33)	0	NA
CXCL2 (ng/ml)	0.05 (0.03 – 0.08)	0	NA
CXCL8 (pg/ml)	1.2 (0.8 – 1.8)	0	NA

BUF: buffer ultrafiltration of the prime; C: complement; CF: complement factor; CCL: C-C motif chemokine ligand; CXCL: C-X-C motif chemokine ligand; CPB: cardiopulmonary bypass; IL: interleukin; NA: not applicable; NR: not recorded; TNF: tumor necrosis factor.

### Inflammatory mediator changes through CPB initiation

**Figure 2.**
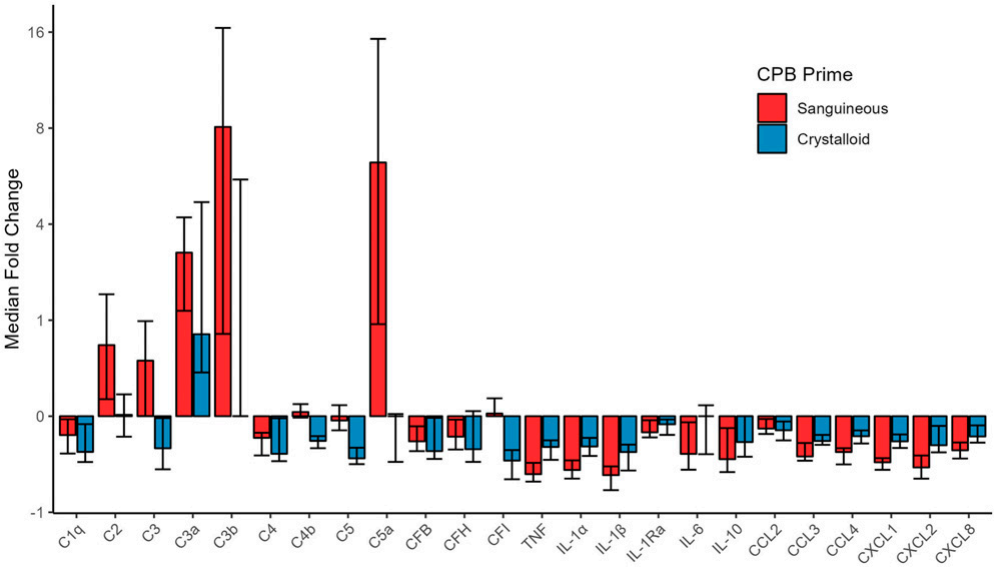
Fold change [95% CI] of circulating inflammatory mediators groups comparing CPB initiation to Pre-CPB baseline values in the patient’s circulation. All patients showed evidence of alternative complement activation upon CPB initiation while only sanguineous prime patients showed downstream complement activity with C5a burden. Cytokines and chemokines were inactive and underwent hemodilution. C: complement; CF: complement factor; CCL: C-C motif chemokine ligand; CXCL: C-X-C motif chemokine ligand; IL: interleukin; TNF: tumor necrosis factor.

**Figure 3.**
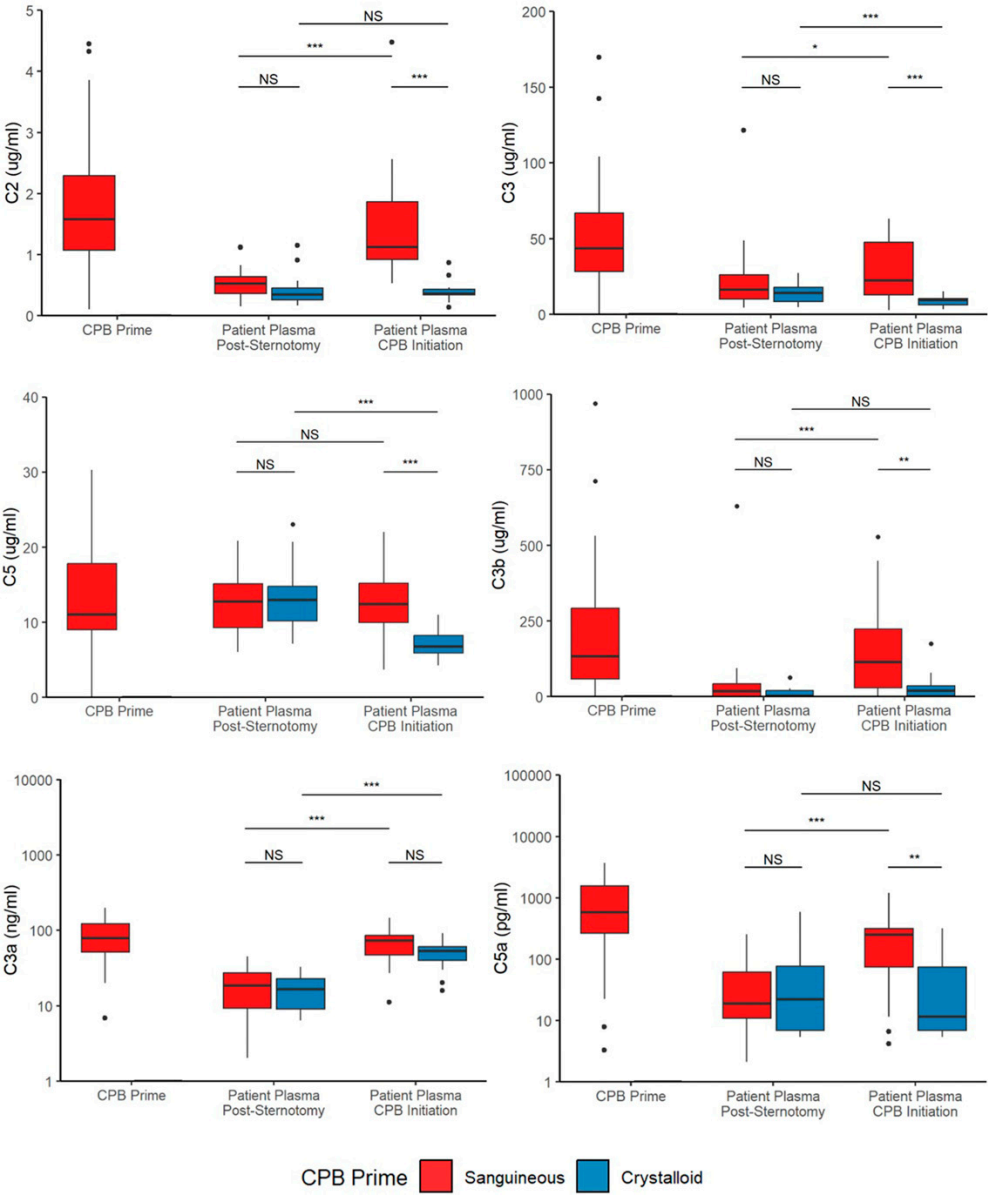
Complement factors are differentially activated between sanguineous and crystalloid CPB prime. Statistical significance levels are denoted: NS indicates no statistical significance (*p* > .05); * for *p* < .05; ** for *p* < .001; and *** for *p* < .0001. *C: complement.*

Upon CPB initiation, the activated complement factors C3a and C3b were elevated in both *sanguineous* and *crystalloid prime* groups while C5a showed significantly higher concentrations in the *sanguineous* group only (Figure 2 & Figure 3). *Sanguineous prime* patients showed dynamic changes of C3a (median fold change of 3.3 [95% CI: 2.1 – 4.2] times), C3b (median fold change of 8.1 [95% CI: 0.9 – 16.5] times) and C5a (median fold change of 6.2 [95% CI: 1.9 – 15.2] times) while the *crystalloid prime* patients showed a more muted response of C3a (median fold change of 1.8 [95% CI: 1.4 – 4.7] times), C3b (median fold change of 0 [95% CI: 0 – 5.5] times) and C5a (median fold change of 0 [95% CI: −0.5 – 0.1] times). The classical complement factors C1q and C4, as well as the regulators CFB and CFH were reduced upon CPB initiation in both groups. C2, C3, C4b, C5 and CFI showed differential profiles between *sanguineous* and *crystalloid prime* groups. The pro-inflammatory cytokines TNF, IL-1α, IL-1β and chemokines CCL2, CCL3, CCL4, CXCL-1, CXCL-2 and CXCL-8 showed significant decreases in both CPB prime groups. IL-6 and the anti-inflammatory mediators IL-1Ra and IL-10 were reduced after CPB initiation in the *sanguineous prime* group but not in the *crystalloid* group. The within-individual differences between CPB initiation and Pre-CPB baseline for both the *sanguineous* and *crystalloid prime* groups as well as the entire cohort are highlighted in Supplement Table S2.

**Figure 4.**
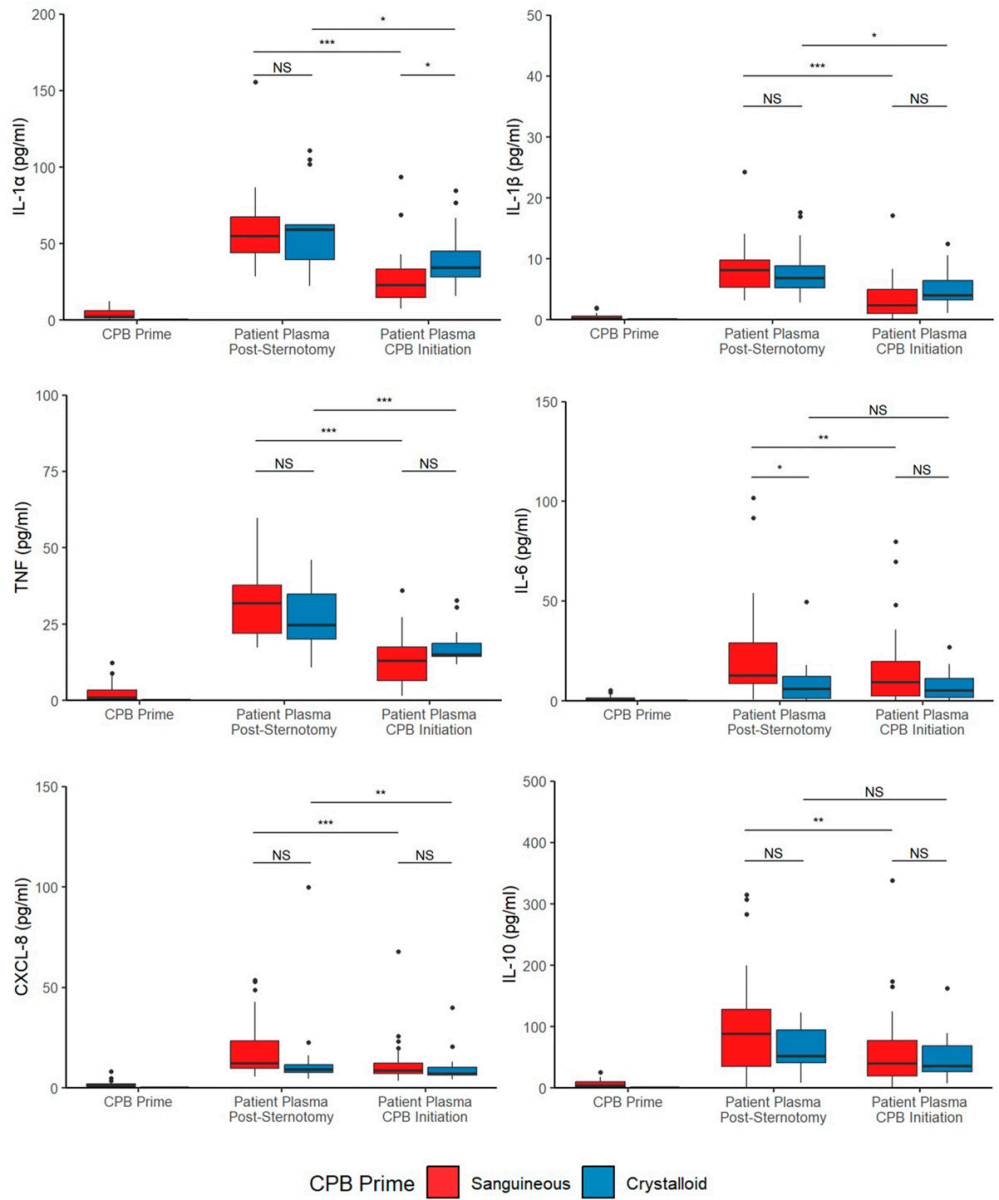
Cytokine and chemokine profiles are stable or decrease in both sanguineous and crystalloid CPB prime. Statistical significance levels are denoted: NS indicates no statistical significance (*p* > .05); * for *p* < .05; ** for *p* < .001; and *** for *p* < .0001. IL: interleukin; TNF: tumor necrosis factor.

Comparing mediator concentrations directly between groups, the *sanguineous prime* patients had statistically higher concentrations of C2 (*p* = 5.8 × 10^−9^), C3 (*p* = 4.0 × 10^−5^), C3b (*p* = 4.0 × 10^−3^), C4b (*p* = 4.7 × 10^−3^), C5 (*p* = 2.5 × 10^−5^), C5a (*p* = 2.6 × 10^−3^) and IL-1Ra (*p* = .01) but lower concentrations of IL-1α (*p* = 5.1 × 10^−3^), IL-1β (*p* = .04), CCL4 (*p* = 6.3 × 10^−4^), CXCL1 (*p* = 2.4 × 10^−3^) and CXCL2 (*p* = 8.3 × 10^−3^) relative to the *crystalloid group* after CPB initiation (Figures 3 and 4 & Supplement Table S3). C1q, C3a, C4, CFB, CFH, CFI, TNF, IL-6, IL-10, CCL2, CCL3 and CXCL8 were comparable between groups.

## Discussion

This study examined the immunologic composition of sanguineous CPB prime and its impact on circulating inflammatory mediators upon CPB initiation relative to a crystalloid CPB prime control. Sanguineous prime contained supraphysiologic concentrations of the complement components C2 and C3 as well as the activated C3a, C3b and C5a. C1q, C4, C4b, C5, CFB, CFH, and CFI were present at levels comparable to the patient’s baseline while inflammatory cytokines and chemokines were detected at trace levels far below that of the patient. This report is the most extensive description to date as only C3a (367 ng/mL), bradykinin (103 pg/mL) and thromboxane B2 (31 pg/mL) have been previously described in sanguineous prime.^14^ Complement factors are known to be found in fresh frozen plasma and the production of anaphylatoxins C3a and C5a are generated through the blood donation, storage and apheresis processes.^18,19^ Both C3a and C5a are extracted by hemoconcentrator devices, but despite BUF treatment of the prime at our institution, these noxious mediators remained present at substantial concentrations.^4^ C3a was found at 79 ng/mL (4.6 times that of the patient baseline) and C5a was measured at 584 pg/mL (17.6 times that of the patient baseline) suggesting robust alternative complement pathway reaction during sanguineous prime preparation. Overall, our results clearly demonstrate reconstituted blood prime in the CPB circuit contains a substantial load of complement substrate, activated components and anaphylatoxins.

The natural next question of these novel findings is to investigate the impact on the patient’s immunology when they are exposed to sanguineous prime. This retrospective study compared two patient groups, with notably different baseline characteristics, but highly similar baseline concentrations of circulating complement factors and cytokines. Upon CPB initiation, patients who received sanguineous prime had markedly higher concentrations of C2, C3, C3b and C5a while C3a was comparable to the control group who received crystalloid prime; the pattern of complement burden is nearly identical to that present in the sanguineous prime apart from C3a. This suggests that the complement found in the sanguinous prime has an immediate and meaningful immunologic influence on the patient. Additionally, these results indicate that the patient’s alternative complement pathway activates rapidly within 5 min of CPB initiation, as seen in the *crystalloid* group’s significant C3a increase from baseline.^2^ Unexpectedly, C3b in the crystalloid group trended towards but did not make a statistically significant increase from baseline. Cytokine and chemokine largely decreased upon CPB initiation in both groups by hemodilution; these non-complement mediators were found in trace amounts in the sanguineous prime and assumed to be absent in crystalloid prime.

Previous investigations have suggested that complement activation may play a role in the pathogenesis of several postoperative complications, such as capillary leak syndrome, ischemia-reperfusion injury and organ dysfunction.^1–3^ However, this hypothesis has not yet been definitively proven. As shown here, sanguineous prime could potentially be a foundational stimulus to the systemic inflammatory response to CPB which features early alternative complement system activation. The classical complement system is known to further enhance the clinically relevant inflammatory response around the time of protamine administration, although those dynamics are not assessed in this current study.^20^ Altogether, these results yield two important questions for future research. First, does the supraphysiologic complement burden in sanguineous prime translate into post-operative morbidities for neonates and infants? If so, what are the dynamics of complement system activation during sanguineous CPB prime preparation and are there therapeutic strategies to inhibit the system? Clearly, this line of inquiry holds many unknowns and potentially a path to dampen inflammation and enhance recovery for these vulnerable patients.

Bloodless prime for small neonatal and infant patients less than 7 kg has been described and is accomplished by minimizing the CPB circuit to volumes ranging between 73 mL and 172 mL.^21,22^ However, a bloodless prime does not mean a transfusion-free surgery. Asanguineous prime for small patients is uncommon and highly dependent on patient selection and institutional expertise; sanguineous prime is most often used.^23,24^ Although miniaturization offers potential to reduce the inflammatory response to the CPB circuit, via reduced surface area and avoidance of allogenic donated blood prime, it has yet to be widely adopted. The potential benefits mitigated by blood product administration during surgery and technical limitations due to the simplified circuit.

This study has important limitations. First, the *sanguineous* and *crystalloid* CPB prime groups are composed of different patients with different baseline characteristics and response to CPB-initiation. Specifically, variables such as age, weight and type of congenital heart disease are known to modulate the inflammatory response to CPB and are unbalanced. Therefore, clinical outcomes cannot be evaluated. For this reason, the analysis focused on mediator changes upon CPB initiation only, as the patient baseline concentrations of these inflammatory factors were comparable. Second, this analysis only examines the inflammatory response at CPB initiation, further longitudinal evaluations are required to understand the immune system’s evolution throughout the CPB time and into the post-operative period where clinical sequelae manifest. This data is available from the prospective trial (NCT05154864) and will be the focus of subsequent investigations. Third, the sample size of the study is small yielding imprecision and asymmetrical confidence intervals in some estimates, particularly in the *crystalloid prime* group. Fourth, this post-hoc study did not assess the presence of bradykinin, lipid inflammatory mediators, or oxidative stress markers from red blood cell storage, all of which could be relevant to the immunologic to sanguineous prime and CPB. Finally, these results are from a single center and generalizability to other institutions with varying perfusion techniques should be made with caution.

In conclusion, despite BUF preparation, sanguineous CPB prime contains a supraphysiologic burden of key complement factors C2, C3, C3a, C3b and C5a. Upon CPB initiation, these inflammatory mediators are directly transferred to neonatal and infant patients, accelerating the complement reaction early in the surgery. Cytokines and chemokines are found in trace concentrations within the sanguineous prime. Future research is required to understand the clinical impact of this phenomenon and to identify effective therapies or CPB prime preparation strategies that can dampen complement activation during pediatric cardiac surgery with CPB.

## Supplemental Material

Supplemental Material - Sanguineous cardiopulmonary bypass prime accelerates the inflammatory response during pediatric cardiac surgery Supplemental Material for Sanguineous cardiopulmonary bypass prime accelerates the inflammatory response during pediatric cardiac surgery by CJoel Bierer, Roger Stanzel, Mark Henderson, John Sapp, Pantelis Andreou, Jean S. Marshall and David Horne Ballard in Perfusion

## Data Availability

The datasets generated and/or analyzed during the current study are not publicly available due to patient data and information confidentiality but are available from the corresponding author on reasonable request.

## Glossary of Abbreviations

BUF: Buffered ultrafiltration of the CPB prime
C: complement
CF: complement factor
CI: confidence interval
CCL: C-C motif chemokine ligand
CXCL: C-X-C motif chemokine ligand
CPB: cardiopulmonary bypass
IL: interleukin
STAT: Society of Thoracic Surgeons-European Association for Cardio-Thoracic Surgery
TNF: tumor necrosis factor
